# Forced Vibration Analysis of Composite Beams Reinforced by Carbon Nanotubes

**DOI:** 10.3390/nano11030571

**Published:** 2021-02-25

**Authors:** Ömer Civalek, Şeref D. Akbaş, Bekir Akgöz, Shahriar Dastjerdi

**Affiliations:** 1 Department of Medical Research, China Medical University Hospital, China Medical University, Taichung 40447, Taiwan; omer@mail.cmuh.org.tw; 2 Department of Civil Engineering, Bursa Technical University, 16310 Bursa, Turkey; seref.akbas@btu.edu.tr; 3 Department of Civil Engineering, Akdeniz University, 07058 Antalya, Turkey; bekirakgoz@akdeniz.edu.tr; 4 Department of Mechanical Engineering, Shahrood Branch, Islamic Azad University, 36199-43189 Shahrood, Iran

**Keywords:** carbon nanotube-reinforced composite, forced vibration, dynamic analysis, beam, harmonic load

## Abstract

This paper presents forced vibration analysis of a simply supported beam made of carbon nanotube-reinforced composite material subjected to a harmonic point load at the midpoint of beam. The composite beam is made of a polymeric matrix and reinforced the single-walled carbon nanotubes with their various distributions. In the beam kinematics, the first-order shear deformation beam theory was used. The governing equations of problem were derived by using the Lagrange procedure. In the solution of the problem, the Ritz method was used, and algebraic polynomials were employed with the trivial functions for the Ritz method. In the solution of the forced vibration problem, the Newmark average acceleration method was applied in the time history. In the numerical examples, the effects of carbon nanotube volume fraction, aspect ratio, and dynamic parameters on the forced vibration response of carbon nanotube-reinforced composite beams are investigated. In addition, some comparison studies were performed, with special results of published papers to validate the using formulations.

## 1. Introduction

Composite material refers to any solid that consists of more than one component, in which they are in separate phases. The main advantages of composite materials are excellent strength-to-weight and stiffness-to-weight ratios. The fibrous composites, consisting of carbon, glass, aramid, and basalt fibers, have a wide range of applications in many modern engineering and industries, such as civil, automotive, bicycle, mechanical, defense, marine, aviation, and aerospace [1,2,3,4,5,6,7,8,9].

In the early 1990s, carbon nanotubes (CNTs) were studied by Sumio Iijima [10]. CNTs can be considered as one of the key building blocks of nanotechnology. Interest of the scientists and researchers in CNTs’ potential engineering and its industrial applications, such as in aerospace, composites, electronics, computers, energy, medicine, sensors, and air and water purifications, have grown rapidly due to the unique material properties of CNTs [11,12,13,14,15,16,17,18].

Due to extraordinary electrical, thermal, and mechanical properties, besides providing good interfacial bonds, CNTs can be ideally suited for the next generation of composite materials and have recently been used instead of the traditional fibers for the reinforcing element in the reinforced composites. Some studies on the bending, buckling, and vibration responses of carbon nanotube-reinforced composite (CNTRC) beams are mentioned below.

Free vibration analysis of functionally graded single-walled CNTs reinforced aluminum alloy beam was performed by Selmi and Bisharat [19]. They obtained the natural frequencies by both the Rayleigh–Ritz method and ANSYS, a software package that implements the finite element method. Pure bending and local buckling behaviors of a nanocomposite beam reinforced by a single-walled carbon nanotube were investigated by Vodenitcharova and Zhang [20]. Ke et al. [21] surveyed the nonlinear free vibration response of functionally graded carbon nanotube-reinforced composite (FG-CNTRC) beams on the basis of first-order shear deformation beam theory with von Kármán geometric nonlinearity assumption. The Ritz method with a direct iterative method was used to obtain solutions for different boundary conditions. Yas and Heshmati [22] investigated the vibrational characteristics of FG-CNTRC beams reinforced by randomly oriented CNTs subjected to a moving load, based on Bernoulli–Euler and Timoshenko beam theories by finite element method. Yas and Samadi [23] analyzed the free vibration and buckling problems of embedded nanocomposite beams resting on an elastic foundation. SWCNTs were used as reinforcement elements. Four different CNTs distributions along the height were taken into consideration. The governing equations were derived by implementing Hamilton’s principle, and the resulting equations were solved by generalized differential quadrature method. Deepak et al. [24] developed a spectral finite element formulation for uniform and tapered rotating CNTRC polymer beams. The obtained results were comparatively presented with the results of carbon fiber-reinforced laminated composite rotating beams. Ke et al. [25] examined the dynamic stability analysis of FG-CNTRC beams subjected to axial loading. The related governing equations were derived based on Timoshenko beam theory and they were solved by differential quadrature method.

Heshmati and Yas [26] perused the free vibration characteristics of FG-CNTRC beams by using an equivalent fiber based on the Eshelby–Mori–Tanaka approach via the finite element method. The influences of agglomeration and distribution of carbon nanotubes were investigated in detail. Wattanasakulpong and Ungbhakorn [27] performed the static bending, free vibration, and buckling analyses of embedded CNTRC beams lying on the Pasternak elastic foundation. Analytic solutions were achieved by Navier’s solution technique for simply supported CNTRC beams. Linear and nonlinear vibrations of CNTRC beams were comprehensively studied on the basis of first and third-order shear deformation beam theories [28,29,30,31,32]. Mayandi and Jeyaraj [33] investigated the static and dynamic behaviors of FG-CNTRC beams subjected to various non-uniform thermal loads by employing the finite element method. Fattahi and Safaei [34] performed stability analysis of CNTRC beams based on three different beam theories. The obtained equations were solved by generalized differential quadrature method. Babu Arumugam et al. [35] surveyed the free and forced vibration responses of CNTRC beams with constant and variable cross-sections by using the finite element method. A detailed parametric work was made to show the influences of slenderness ratio, percentage of CNT constituent, ply orientation, and boundary conditions. Mohseni and Shakouri [36] studied the free vibration and buckling responses of tapered FG-CNTRC beams surrounding an elastic medium based on Timoshenko beam theory. Dynamic analysis of the pre-twisted FG-CNTRC beams subjected to thermal loading was carried out by Shenas et al. [37]. A higher-order shear deformation beam theory was employed to derive the constitutive equations, and the Chebyshev–Ritz method was applied to solve the resulting equations for various end conditions. Khosravi et al. [38] performed the thermal stability analysis of rotating CNTRC beams. Timoshenko beam theory was used in the derivation of the governing differential equations. Generalized differential quadrature method was employed to obtain some numerical results. Additionally, buckling and vibration responses of micro-and nano-composite beams were investigated [39,40,41]. On the other hand, there have recently been many studies on the mechanical behaviors of CNTRC plates and shells [42,43,44,45,46,47,48,49,50,51,52,53,54,55,56].

As mentioned above, such structures may be subjected to various loads. Due to this fact, it is very important to understand the dynamical behavior of these structures subjected to the harmonic loads. Additionally, the aforementioned review reveals that researchers have so far examined the bending, buckling, and free vibration responses of CNTRC beams. In particular, to the best of the authors’ knowledge, forced vibration of CNTRC beams due to harmonic loads has not been investigated in detail. The main purpose of the present study is to fill this gap.

In this paper, forced vibrational behavior of CNTRC beam is examined. It was considered that CNTRC beams are made of a polymeric matrix reinforced by the single-walled carbon nanotubes and is subjected to a harmonic point load at the middle. Three different distributions of CNTs are considered in the analysis. The governing equations of problem have been derived by using the Lagrange procedure based on Timoshenko beam theory. The Ritz method, in conjunction with algebraic polynomials with the trivial functions, was utilized to solve the resulting equation. Additionally, the Newmark average acceleration method was used in the time history for the solution of the forced vibration problem. A detailed parametric study was carried out to peruse the influences of CNTs volume fraction, slenderness ratio, and dynamic parameters on the forced vibration response of CNTRC beam.

## 2. Theory and Formulation

**Figure 1:**
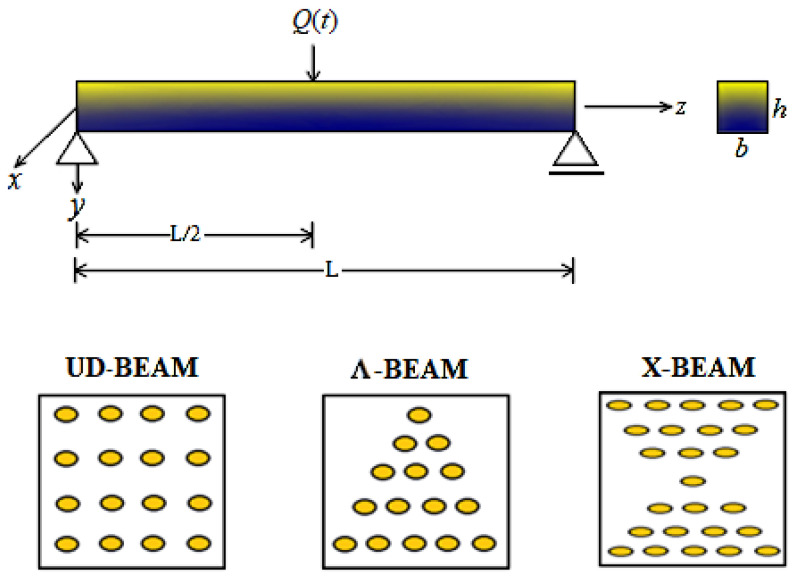
A schematic illustration of a simply supported carbon nanotube-reinforced composite (CNTRC) beam under a harmonic load and three different patterns of CNTs.

Consider a simply supported beam made of CNTRC material under a dynamic load, as shown in Figure 1. The composite beam was subjected to a dynamic point load, Q(t), at the midpoint. The geometry of the beam was indicated as the length, *L*; the height, *h*; and width, *b*. Additionally, three different patterns of CNTs, such as uniformly distributed (UD), Ʌ- and X- type distributions, were considered throughout the thickness of the composite beam.

The dynamic point load (Q(t)) was assumed to be sinusoidal harmonic in time domains, such as in the following:

(1) $$\left(t\right)=Q_{0}\sin\left(\bar{\omega }t\right),\;0\leq t\ll \infty$$

where $Q_{0}$ and $\bar{\omega }$ denote the amplitude and frequency of the dynamic load, respectively.

The effective material properties for CNTRC beams are given below [27,57]:

(2) $$E_{11}=\eta_{1}V_{CNT}E_{11}^{CNT}+V_{p}E^{p}$$

(3) $$\frac{\eta_{2}}{E_{22}}=\frac{V_{CNT}}{E_{22}^{CNT}}+\frac{V_{p}}{E^{p}}$$

(4) $$\frac{\eta_{3}}{G_{12}}=\frac{V_{CNT}}{G_{12}^{CNT}}+\frac{V_{p}}{G^{p}}$$

(5) $$V_{CNT}+V_{p}=1$$

(6) $$v=V_{CNT}v^{CNT}+V_{p}v^{p}$$

(7) $$\rho =V_{CNT}\rho^{CNT}+V_{p}\rho^{p}$$

where E, G, v, and ρ are the material properties that represent the Young’s modulus of elasticity, shear modulus, Poisson’s ratio, and density, respectively. The superscripts of CNT and p respectively symbolize the related material properties of the carbon nanotube and polymer matrix. $\eta_{1},\;\eta_{2},\eta_{3}$ can be indicated the efficiency parameters of CNT. Additionally, *V_CNT_* and *V_p_* define the volume fractions for CNT and polymer matrix, respectively.

The axial strain ($\varepsilon_{z}$) and shear strain ($\gamma_{zy}$) are given according to the first-order shear deformation beam theory, as follows:

(8a) $$\varepsilon_{z\;=}\frac{\partial u_{0}}{\partial z}-Y\frac{\partial \emptyset }{\partial z}$$

(8b) $$\gamma_{zy}=\frac{\partial u_{0}}{\partial y}+\frac{\partial v_{0}}{\partial z}$$

where $u_{0}$, $v_{0}$, and ∅ are axial and vertical displacements, and rotation, respectively. The constitute relation is given below:

(9a) $$\sigma_{z}=E\left(Y\right)\left[\frac{\partial u_{0}}{\partial z}-Y\frac{\partial \emptyset }{\partial z}\right]$$

(9b) $$\sigma_{zy}=G\left(Y\right)K_{s}\left[\frac{\partial v_{0}}{\partial z}-\emptyset \right]$$

where *E* is the Young’s modulus, G is the shear modulus, $\sigma_{z}$ is the normal stress, $\sigma_{zy}$ is the shear stress, and $K_{s}$ is the shear correction factor.

The strain energy (*U*_i_), the kinetic energy (*K*), the dissipation function, and potential energy of the external loads (*U*_e_) are presented as follows:

(10a) $$U_{i}=\frac{1}{2}\int_{0}^{L}\left[A_{0}{\left(\frac{\partial u_{0}}{\partial z}\right)}^{2}-2A_{1}\frac{\partial u_{0}}{\partial z}\frac{\partial \emptyset }{\partial z}+A_{2}{\left(\frac{\partial \emptyset }{\partial z}\right)}^{2}\right]dZ+\;\frac{1}{2}\int_{0}^{L}K_{s}B_{0}\left[{\left(\frac{\partial v_{0}}{\partial z}\right)}^{2}-2\frac{\partial v_{0}}{\partial z}\emptyset +\emptyset^{2}\right]dZ$$

(10b) $$K=\frac{1}{2}\int_{0}^{L}\left(I_{0}{\left(\frac{\partial u_{0}}{\partial t}\right)}^{2}-2I_{1}\left(\frac{\partial u_{0}}{\partial t}\right)\left(\frac{\partial \emptyset }{\partial t}\right)+I_{2}{\left(\frac{\partial \emptyset }{\partial t}\right)}^{2}+I_{0}{\left(\frac{\partial v_{0}}{\partial t}\right)}^{2}\right)dZ$$

(10c) $$U_{e}=-Q\left(t\right)\;v\left(z_{p},t\right)$$

where

(11a) $$\left(A_{0},\;A_{1},A_{2}\right)=\overset{}{\underset{A}{\int }}E\left(Y\right)\left(1,Y,Y^{2}\right)dA,\;$$

(11b) $$\;B_{0}=\underset{A}{\int }G\left(Y\right)dA$$

(11c) $$\left(I_{0},I_{1},I_{2}\right)=\overset{}{\underset{A}{\int }}\rho \left(Y\right)\left(1,Y,Y^{2}\right)dA$$

The Lagrangian functional of the problem is presented as:

(12) $$I=K-\left(U_{i}+U_{e}\right)$$

In the solution of the problem in the Ritz method, the approximate solution is given as a series of *i* terms, as in the following:

(13a) $$u_{0}\left(z,t\right)=\;\overset{\infty }{\underset{i=1}{\sum }}a_{i\;}\left(t\right)\alpha_{i}\left(z\right)$$

(13b) $$v_{0}\left(z,t\right)=\;\overset{\infty }{\underset{i=1}{\sum }}b_{i\;}\left(t\right)\beta_{i}\left(z\right)$$

(13c) $$\emptyset \left(z,t\right)=\;\overset{\infty }{\underset{i=1}{\sum }}c_{i\;}\left(t\right)\gamma_{i}\left(z\right)$$

where *a_i_*, *b_i_*, and *c_i_* are the unknown coefficients, and $\alpha_{i}\left(z,t\right)$, $\beta_{i}\left(z,t\right)$, and $\gamma_{i}\left(z,t\right)$ are the coordinate functions depending on the end conditions over the interval [*0*, *L*]. The coordinate functions for the simply supported beam are given as algebraic polynomials:

(14a) $$\alpha_{i}\left(z\right)=z^{i}$$

(14b) $$\beta_{i}\left(z\right)=\left(L-z\right)z^{i}$$

(14c) $$\gamma_{i}\left(z\right)=z^{\left(i-1\right)}$$

where *i* indicates the number of polynomials involved in the admissible functions. After substituting Equation (7) into Equation (4), and then using the Lagrange’s equation, the following equation can be derived:

(15) $$\frac{\partial I}{\partial q_{i}^{}}-\frac{\partial }{\partial t}\frac{\partial I}{\partial {\dot{q}}_{i}^{}}=0$$

where *q_i_* is the unknown coefficients which are *a_i_*, *b_i_*, and *c_i_*. After implementing the Lagrange procedure, the motion equation of the problem was obtained as follows:

(16) $$\left[K\right]\left\{q\left(t\right)\right\}+\left[M\right]\left\{\ddot{q}\left(t\right)\right\}=\left\{F\left(t\right)\right\}$$

where [K], [M], and {F(t)} are the stiffness matrix, mass matrix, and load vector, respectively. The details of these expressions are given as follows:

(17) $$\left[K\right]=\left[\begin{array}{ccc} K_{11} & K_{12} & K_{13}\\ K_{21} & K_{22} & K_{23}\\ K_{31} & K_{32} & K_{33} \end{array}\right]$$

where

(18a) $$K_{ij}^{11}=\overset{n}{\underset{i=1}{\sum }}\overset{n}{\underset{j=1}{\sum }}\int_{0}^{L}A_{0}\frac{\partial \alpha_{i}}{\partial z}\frac{\partial \alpha_{j}}{\partial z}dz$$

(18b) $$K_{ij}^{12}=0,$$

(18c) $$K_{ij}^{13}\;=-\overset{n}{\underset{i=1}{\sum }}\overset{n}{\underset{j=1}{\sum }}\int_{0}^{L}A_{1}\frac{\partial \alpha_{i}}{\partial z}\frac{\partial \gamma_{j}}{\partial z}dz$$

(18d) $$K_{ij}^{21}=0,$$

(18e) $$K_{ij}^{22}=\;\overset{n}{\underset{i=1}{\sum }}\overset{n}{\underset{j=1}{\sum }}\int_{0}^{L}K_{s}B_{0}\frac{\partial \beta_{i}}{\partial z}\frac{\partial \beta_{j}}{\partial z}dz$$

(18f) $$K_{ij}^{23}=-\overset{n}{\underset{i=1}{\sum }}\overset{n}{\underset{j=1}{\sum }}\int_{0}^{L}K_{s}B_{0}\frac{\partial \beta_{i}}{\partial z}\gamma_{j}dz$$

(18g) $$K_{ij}^{31}=-\overset{n}{\underset{i=1}{\sum }}\overset{n}{\underset{j=1}{\sum }}\int_{0}^{L}A_{1}\frac{\partial \gamma_{i}}{\partial z}\frac{\partial \alpha_{j}}{\partial z}dz$$

(18h) $$K_{ij}^{32}=-\overset{n}{\underset{i=1}{\sum }}\overset{n}{\underset{j=1}{\sum }}\int_{0}^{L}K_{s}B_{0}\gamma_{i}\frac{\partial \beta_{j}}{\partial z}dz$$

(18i) $$K_{ij}^{33}=\overset{n}{\underset{i=1}{\sum }}\overset{n}{\underset{j=1}{\sum }}\int_{0}^{L}A_{2}\frac{\partial \gamma_{i}}{\partial z}\frac{\partial \gamma_{j}}{\partial z}+\overset{n}{\underset{i=1}{\sum }}\overset{n}{\underset{j=1}{\sum }}\int_{0}^{L}K_{s}B_{0}\gamma_{i}\gamma_{j}dz$$

(19) $$\left[M\right]=\left[\begin{array}{ccc} M_{11} & M_{12} & M_{13}\\ M_{21} & M_{22} & M_{23}\\ M_{31} & M_{32} & M_{33} \end{array}\right]$$

where

(20a) $$M_{11}=\overset{n}{\underset{i=1}{\sum }}\overset{n}{\underset{j=1}{\sum }}\overset{L}{\underset{0}{\int }}I_{0}\alpha_{i}\alpha_{j}dz$$

(20b) $$M_{12}=0$$

(20c) $$M_{13}=-\overset{n}{\underset{i=1}{\sum }}\overset{n}{\underset{j=1}{\sum }}\overset{L}{\underset{0}{\int }}I_{1}\alpha_{i}\gamma_{j}dz$$

(20d) $$M_{21}=0$$

(20e) $$M_{22}=\overset{n}{\underset{i=1}{\sum }}\overset{n}{\underset{j=1}{\sum }}\overset{L}{\underset{0}{\int }}I_{0}\beta_{i}\beta_{j}dz$$

(20f) $$M_{23}=0$$

(20g) $$M_{31}=-\overset{n}{\underset{i=1}{\sum }}\overset{n}{\underset{j=1}{\sum }}\overset{L}{\underset{0}{\int }}I_{1}\gamma_{i}\alpha_{j}dz$$

(20h) $$M_{32}=0$$

(20i) $$M_{33}=\overset{n}{\underset{i=1}{\sum }}\overset{n}{\underset{j=1}{\sum }}\overset{L}{\underset{0}{\int }}I_{2}\gamma_{i}\gamma_{j}dz$$

(21) $$\left\{F\left(t\right)\right\}=Q\beta_{j}$$

The constitutive equation of motions was solved by implementing implicit Newmark average acceleration method with α=0.5 and β=0.25 in the time domain. By this procedure, the dynamic problem will be transferred to a system of static problems in each step, as in the following:

(22) $$\left[\bar{K}\left(t,X\right)\right]{\left\{d_{n}\right\}}_{j+1}=\left\{\bar{F}\left(t\right)\right\}$$

in which

(23a) $$\left[\bar{K}\left(t,X\right)\right]=\left[K\right]+\frac{\left[M\right]}{\beta \Delta t^{2}}+\frac{\left[C\right]\alpha }{\beta \Delta t}$$

(23b) $$\left\{\bar{F}\left(t\right)\right\}={\left\{F\left(t\right)\right\}}_{j+1}+B_{1}{\left\{d_{n}\right\}}_{j}+B_{2}{\left\{{\dot{d}}_{n}\right\}}_{j}+B_{3}{\left\{{\ddot{d}}_{n}\right\}}_{j}$$

where

(24) $$B_{1}=\frac{\left[M\right]}{\beta \Delta t^{2}},\;B_{2}=\frac{\left[M\right]}{\beta \Delta t},\;B_{3}=\left[M\right]\left(\frac{1}{2\beta }-1\right)$$

After evaluating ${\left\{d_{n}\right\}}_{j+1}$ at a time $t_{j+1}=t_{j}+\Delta t$, the acceleration and velocity vectors can be expressed as:

(25a) $${\left\{{\ddot{d}}_{n}\right\}}_{j+1}=\frac{1}{\beta \Delta t^{2}}\left({\left\{d_{n}\right\}}_{j+1}-{\left\{d_{n}\right\}}_{j}\right)-\frac{\left[M\right]}{\beta \Delta t}{\left\{{\dot{d}}_{n}\right\}}_{j}-\left(\frac{\alpha }{2\beta }-1\right){\left\{{\ddot{d}}_{n}\right\}}_{j}$$

(25b) $${\left\{{\dot{d}}_{n}\right\}}_{j+1}={\left\{{\dot{d}}_{n}\right\}}_{j}+\Delta t\;\left(1-\alpha \right){\left\{{\ddot{d}}_{n}\right\}}_{j}+\Delta t\;\alpha \;{\left\{{\ddot{d}}_{n}\right\}}_{j+1}$$

## 3. Numerical Results

In the numerical study, the effects of carbon nanotube volume fraction, aspect ratio, and dynamic parameters on the forced vibration response of CNTRC beams are presented and discussed. The five-point Gauss rule was employed to calculate the integration. In the numerical results, the number of terms is taken as 10. Volume fractions of CNTs as functions of thickness direction for different patterns of CNTs [27] are presented in Table 1. In this table, $V_{CNT}^{*}$ is the given volume fraction of CNTs.

In this study, it is notable that CNTs are parallel to the longitudinal direction of the composite beam. Additionally, the efficiency parameters of CNTs for three different values of $V_{CNT}^{*}$ were considered as [23]:

(26a) $$\eta_{1}=1.2833,\;\eta_{2}=\eta_{3}=1.0556\;\mathrm{for}\;V_{CNT}^{*}=0.12$$

(26b) $$\eta_{1}=1.3414,\;\eta_{2}=\eta_{3}=1.7101\;\mathrm{for}\;V_{CNT}^{*}=0.17$$

(26c) $$\eta_{1}=1.3238,\;\eta_{2}=\eta_{3}=1.7380\;\mathrm{for}\;V_{CNT}^{*}=0.28$$

In the numerical results, the following dimensionless displacement was used:

(27) $$\bar{v}=\frac{E^{p}bh^{3}}{PL^{3}}v$$

In the present analysis, the material properties for reinforcement and matrix constituents were [23,27]: $E_{11}^{CNT}=600\;GPa$, $E_{22}^{CNT}=10\;GPa$, $G_{12}^{CNT}=17.2\;GPa$, $v^{CNT}=0.19$, $\rho^{CNT}=1400\;kg/m^{3}$, $E^{p}=2.5\;GPa$, $v^{p}=0.30$, and $\rho^{p}=1190\;kg/m^{3}$.

In order to validate the present formulations and analyses, some comparative results are listed in Table 2 and Table 3. Firstly, a comparison of non-dimensional fundamental frequencies ($\lambda =\frac{L^{2}}{h}/\sqrt{\rho_{a}/E_{a}}$) of simply supported functionally graded CNT/Aluminum (Al)-alloy composite beams with ANSYS results [19] is presented in Table 2. Here, *k* is the power-law index, and $E_{a}$ and $\rho_{a}$ represent the elastic modulus and density of pure Al-alloy material, respectively. It can be observed (according to Table 2) that the present results agree well with the ANSYS results [19]. The dimensionless fundamental frequencies ($\omega_{b}=\omega L/\sqrt{I_{0}/A_{0}}$) of simply supported CNTRC beams were calculated with different volume fractions of CNTs for *L/h* = 15 and *V_CNT_* = 0.12 compared with those of Wattanasakulpong and Ungbhakorn [27], corresponding to the first-order shear deformation theory. To obtain the vibration frequency in this study, the eigenvalue process is implemented in Equation (16). It is seen from Table 3 that the present results are in good agreement with that of the results of Wattanasakulpong and Ungbhakorn [27].

**Figure 2:**
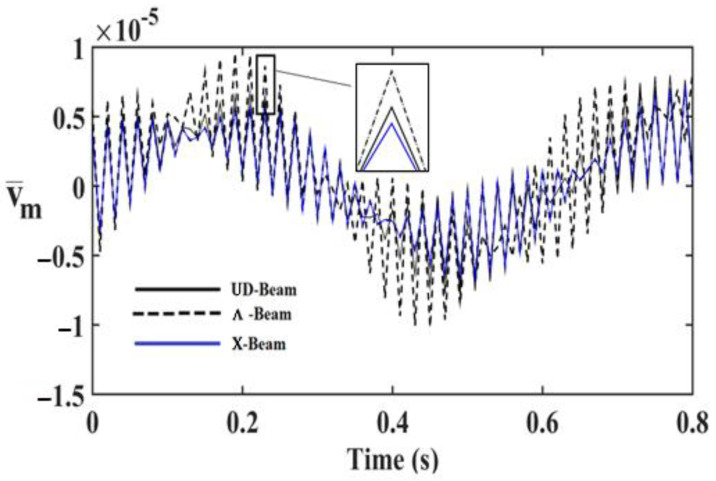
Time responses of the CNTRC beam with different reinforcement patterns for *V_CNT_* = 0.12.

**Figure 3:**
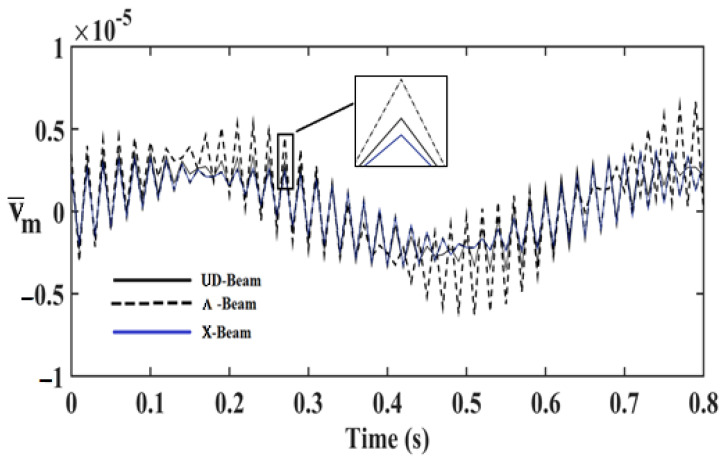
Time responses of the CNTRC beam with different reinforcement patterns for *V_CNT_* = 0.17.

**Figure 4:**
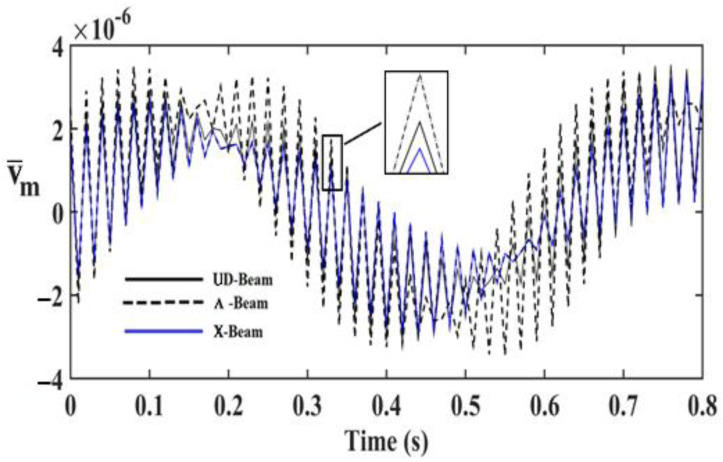
Time −responses of the CNTRC beam with different reinforcement pattern for *V_CNT_* = 0.28.

**Figure 5:**
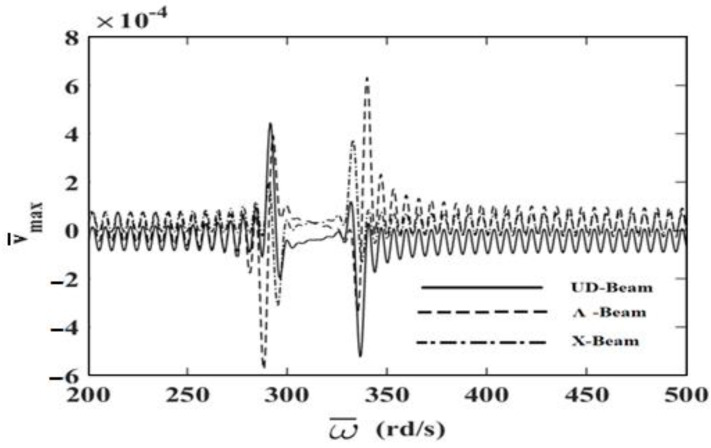
The relationship between the displacements and the frequency of the dynamic load ($\bar{w}$) for *V_CNT_* = 0.12.

**Figure 6:**
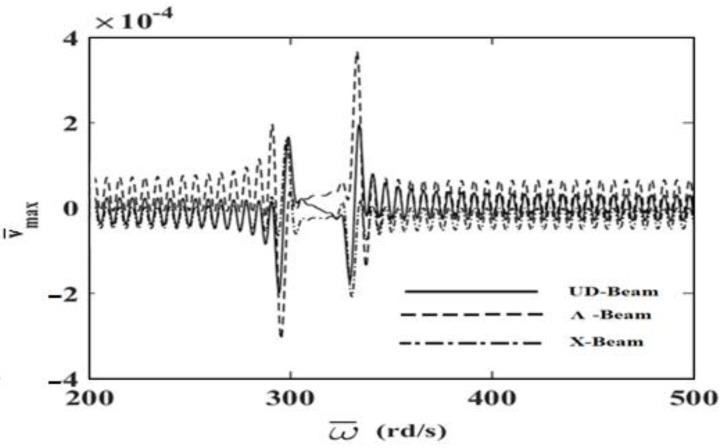
The relationship between the displacements and the frequency of the dynamic load ($\bar{w}$) for *V_CNT_* = 0.17.

**Figure 7:**
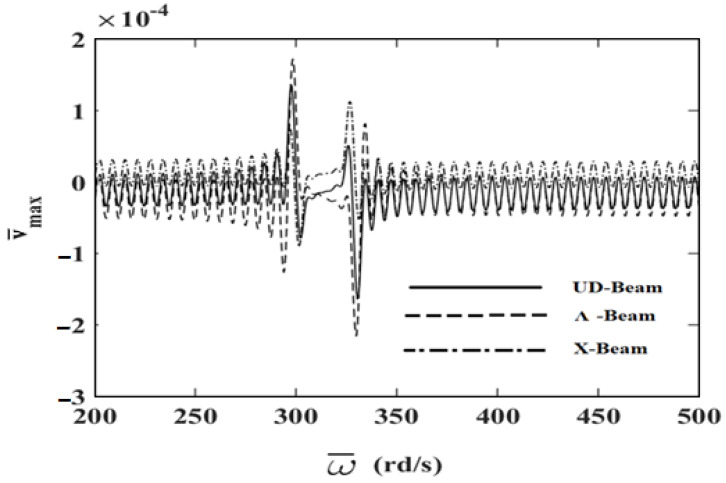
The relationship between the displacements and the frequency of the dynamic load ($\bar{w}$) for *V_CNT_* = 0.28.

In order to investigate the effects and compare different reinforcement patterns on dynamic responses, time responses of the simply supported CNTRC beams are presented in Figure 2, Figure 3 and Figure 4 for volume fractions of CNTs of *V_CNT_* = 0.12, *V_CNT_* = 0.17, and *V_CNT_* = 0.28, respectively. In these figures, the dimensionless midpoint displacements (${\bar{v}}_{m}$) of the beam are obtained within time history for aspect ratio *L/h* = 7 and the external load frequency $\bar{\omega }=10\;rd/s$. In addition, the dynamical dimensionless displacements of the midpoint (${\bar{v}}_{m}$) and the frequency of the dynamic load ($\bar{w}$) relations are presented for different reinforcement patterns for *L/h* = 10 and *t* = 1 s in Figure 5, Figure 6 and Figure 7 for volume fractions of CNTs *V_CNT_* = 0.12, *V_CNT_* = 0.17, and *V_CNT_* = 0.28.

**Figure 8:**
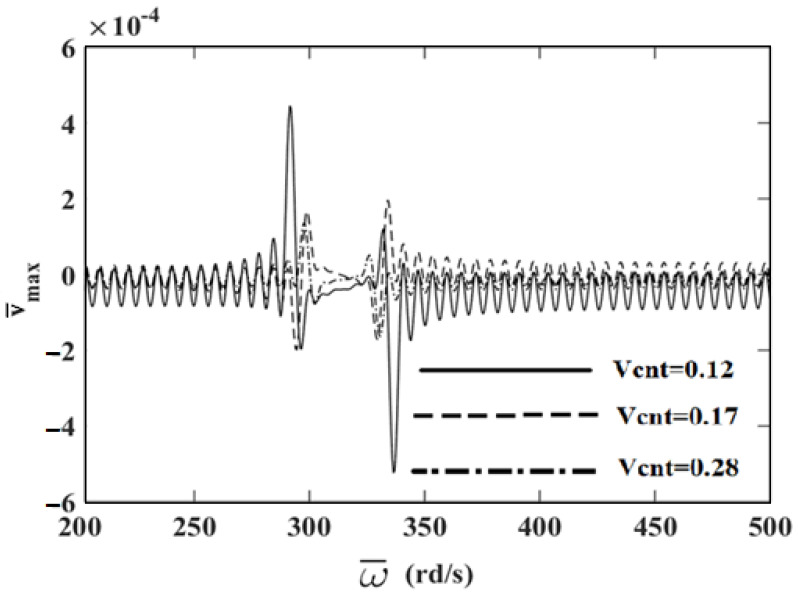
The relationship between the displacements and the frequency of the dynamic load ($\bar{w}$) in the simply supported uniformly distributed (UD)-beam for different values of *V_CNT_*.

**Figure 9:**
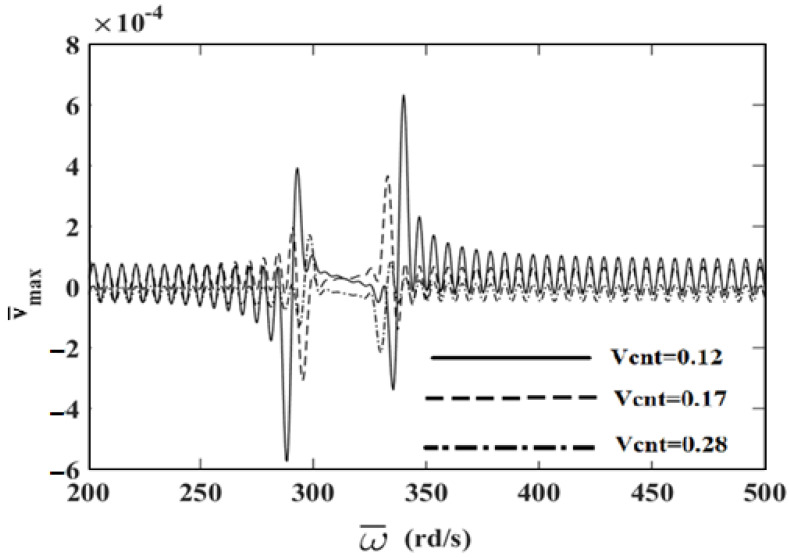
The relationship between the displacements and the frequency of the dynamic load ($\bar{w}$) in the simply supported Λ-Beam for different values of *V_CNT_*.

**Figure 10:**
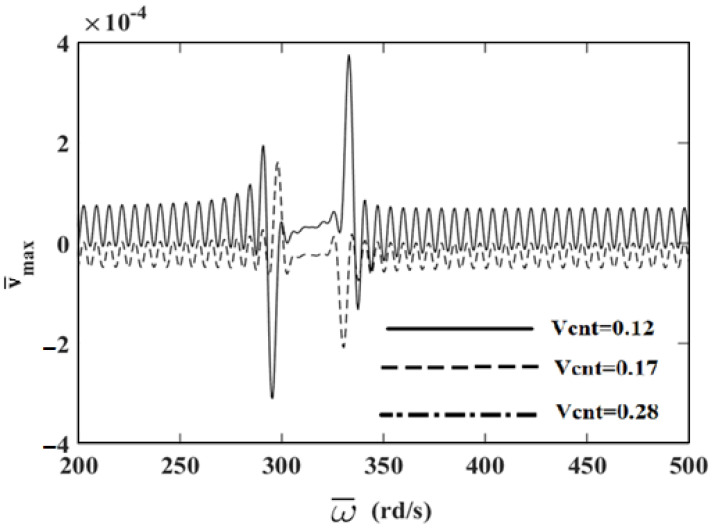
The relationship between of the displacements and the frequency of the dynamic load ($\bar{w}$) in the simply supported X-Beam for different values of *V_CNT_*.

It is shown, according to Figure 2, Figure 3, Figure 4, Figure 5, Figure 6, Figure 7, Figure 8, Figure 9 and Figure 10, that the dynamical displacements of the Ʌ-Beam are the biggest of all. In Ʌ-Beam, the reinforcements spread only at the bottom surface, not upper surface. However, the reinforcements spread at both surfaces on the UD-Beam and X-Beam. It was known that the upper and lower surfaces of the beam have high stresses and strains. Therefore, the Ʌ-Beam model had the lowest rigidity in all models. As a result, more displacements occured in the Ʌ-Beam model. This situation could be observed in Table 3. The vibration frequency of the Ʌ-Beam was lower than the frequency of the other models. Additionally, the dynamical displacements of the UD-Beam were bigger than those of the X-beam. This is because of the reinforcements spread at both surfaces in the UD- and X-Beams, the X-beam has the biggest specific strength in all patterns. Therefore, dynamic response of the X-Beam is lower than all.

Influence of volume fractions of CNTs on the resonance frequencies of the reinforced composite beam with different distribution patterns are revealed in Table 4. It was found that an increase in volume fractions of CNTs gives rise to an increment in resonance frequencies. Additionally, the highest resonance frequencies occur in the X-Beam, while the lowest ones occur in Ʌ-Beam.

In Figure 5, Figure 6 and Figure 7, the resonance phenomenon can be observed in the vertical asymptote regions. In the Ʌ-Beam, the resonance frequency is the lowest for all reinforcement distribution models, because the rigidity of the Ʌ-Beam is lowest for all. Increasing the volume fractions of CNTs (*V_CNT_*) yields increased resonance frequency and decreased displacements. It can be interpreted that, by increasing the volume fractions of CNTs, the beam gets more strength. Therefore, the resonance frequencies increase and dynamically displacements decrease naturally.

Figure 8, Figure 9 and Figure 10 display the frequency of the dynamic load ($\bar{w}$)-dimensionless vertical displacements relationship for *L/h* = 10 and *t* = 1 *s* for different values of *V_CNT_* for the UD-beam, Ʌ-Beam, and X-Beam. It was observed that the increase in values of *V_CNT_* cause a decrease in the amplitudes of displacements. In the X-beam, the resonance frequencies obtained were the lowest values, in contrast with other values of reinforcement patterns. Another result is that the difference among values of *V_CNT_* is the highest in the Ʌ-Beam. It can be concluded that the effects of volume fractions of CNTs were more effective in Ʌ-Beams. It shows that the distribution of the reinforcement plays an important role on dynamic responses of CNTRC beams.

## 4. Conclusions

The forced vibration response of a simply supported CNTRC beam subjected to a harmonic point load was investigated. It was considered that the composite beam was composed of a polymeric matrix (Poly methyl methacrylate) and a reinforcing material (single-walled carbon nanotubes). Timoshenko beam theory was employed in order to take into consideration the effects of shear deformation. The Ritz and Newmark average acceleration methods were used to obtain the numerical results. The effects of volume fraction and distribution patterns of CNTs, aspect ratio, and dynamic parameters on the forced vibration behavior of CNTRC beam were investigated in detail. It was observed that the greatest dynamical displacements occurred in the Ʌ-Beam, dependent on having the smallest rigidity. Additionally, it was found that the lowest resonance frequencies were obtained in the X-Beam. In addition, it was revealed that an increase in the values of V_CNT_ gave rise to a decrement in the amplitudes of displacements. Moreover, it was emphasized that the distribution pattern of the reinforcement plays an important role on dynamic responses of CNTRC beams.

## Figures and Tables

**Table 1:** Volume fractions of CNTs as a function of thickness direction for different distributions of CNTs [27].

Patterns of CNTs	$V_{CNT}$
UD	$V_{CNT}^{*}$
FG-V	$V_{CNT}^{*}\left(1+2\frac{z}{h}\right)$
FG-Λ	$V_{CNT}^{*}\left(1-2\frac{z}{h}\right)$
FG-O	$2V_{CNT}^{*}\left(1-2\frac{\left|z\right|}{h}\right)$
FG-X	$4V_{CNT}^{*}\frac{\left|z\right|}{h}$

**Table 2:** Comparison of non-dimensional fundamental frequencies of simply supported functionally graded CNT/Al-alloy composite beams with ANSYS results.

k	0	0.4	1	2	5	10	Al-Alloy
ANSYS [19]	3.4668	3.2718	3.1496	3.0795	3.0084	2.9546	2.8500
Present	3.663	3.459	3.342	3.271	3.193	3.134	2.971

**Table 3:** Comparative results for dimensionless fundamental frequencies of a simply supported CNTRC beam for *L/h* = 15, *V_CNT_* = 0.12.

Volume Fractions of CNTs	Present	Wattanasakulpong and Ungbhakorn [27]
UD-Beam	0.9905	0.9976
Ʌ-Beam	0.8562	0.8592
X-Beam	1.1373	1.1485

**Table 4:** The resonance frequencies of reinforced composite beams for various volume fractions of CNTs (*L* = 2 m, *b* = *h* = 0.1 m).

V_CNT_	$\bar{w}$ (rd/s)		
	UD-Beam	Ʌ-Beam	X-Beam
0.12	592.98	445.39	692.32
0.17	723.80	538.75	850.10
0.28	881.36	664.42	1028.80

## Data Availability

Data sharing not applicable.
